# MATLAB-candexch algorithm-enhanced UV spectrophotometric-chemometric models for green, blue, and white determination of cinnarizine, domperidone, and carcinogenic impurity in pharmaceuticals: NQS assessment and UN-SDGs integration

**DOI:** 10.1186/s13065-026-01779-0

**Published:** 2026-04-12

**Authors:** Hussein N. Ghanem, Asmaa A. El-Zaher, Enas A. Taha, Ahmed Emad F. Abbas, Sally T. Mahmoud

**Affiliations:** 1 Chemistry Department, Faculty of Pharmacy, October 6 University, 6 October City, Giza, 12585 Egypt; 2 Pharmaceutical Chemistry Department, Faculty of Pharmacy, Cairo University, Kasr El-Aini St., Cairo, 11562 Egypt; 3 Analytical Chemistry Department, Faculty of Pharmacy, October 6 University, 6 October City, Giza, 12585 Egypt; 4 Pharmaceutical Chemistry Department, School of Pharmacy, New Giza University, New Giza, km 22 Cairo– Alexandria Desert Road, Cairo, Egypt

**Keywords:** UV spectrophotometry, Chemometric models, D-optimal design, Pharmaceutical analysis, Sustainability assessment

## Abstract

Novel analytical methodologies integrating UV spectrophotometric techniques with chemometric models were developed for the simultaneous determination of cinnarizine (CIN), domperidone (DOM), and benzophenone (BNZ), a carcinogenic degradation product of CIN, without prior separation. Despite their clinical significance, no existing methods have been reported for their simultaneous quantification. This approach aligns with green and white analytical chemistry principles, offering an environmentally sustainable solution. Predictive models were constructed using Classical Least Squares (CLS), Partial Least Squares (PLS), and Multivariate Curve Resolution-Alternating Least Squares (MCR-ALS). A key methodological advancement was the application of D-optimal design via MATLAB’s Candexch algorithm, generating a strategically balanced validation dataset of 13 mixtures and overcoming limits of conventional data splitting in chemometric modelling. The methods demonstrated robust linearity across concentration ranges of 4–20 µg/mL for CIN, 3–15 µg/mL for DOM, and 1–5 µg/mL for BNZ. Calibration performance was excellent, with root mean square errors of calibration (RMSEC) values of 0.036–0.062 for CLS, 0.013–0.024 for PLS, and 0.009–0.012 for MCR-ALS. Notably, MCR-ALS exhibited the smallest and most consistent RMSEC range, demonstrating superior accuracy and stability compared to other chemometric models. Validation studies confirmed excellent method performance with recovery rates between 98 and 102%. The root mean square errors of prediction (RMSEP) for the validation set were 0.042–0.201 for CLS, 0.035–0.187 for PLS, and 0.022–0.154 for MCR-ALS, with MCR-ALS consistently exhibiting exceptional predictive capability. The environmental sustainability credentials of the methodology were comprehensively evaluated using nine distinct evaluation tools: NEMI, Complex GAPI, AGREE, BAGI, RGB12, SDAGI, Carbon Footprint Analysis, GSST, and NQS. These rigorous assessments confirmed the method's exceptional environmental compatibility while maintaining analytical excellence, positioning this approach as an ideal sustainable alternative for pharmaceutical quality control applications aligned with UN Sustainable Development Goals.

**Supplementary Information:**

The online version contains supplementary material available at 10.1186/s13065-026-01779-0.

## Introduction

Analytical chemistry faces an increasing challenge to develop methodologies that balance analytical performance with environmental sustainability, cost-effectiveness, and practical applicability. This challenge is particularly acute in pharmaceutical analysis, where high precision and accuracy must be achieved without compromising environmental responsibility [1]. In response to these demands, the scientific community has embraced the fundamentals of White Analytical Chemistry (WAC) and Green Analytical Chemistry (GAC), which emphasize minimizing environmental impact while maintaining or improving analytical efficiency [2–5].

To systematically evaluate and improve the sustainability of analytical methods, researchers have developed numerous assessment tools, including the Green Solvent Selection Tool (GSST) [6], Spider Diagram for Assessment of the Greenness Index (SDAGI) [7], National Environmental Method Index (NEMI) [8], Complementary Green Analytical Procedure Index (Complex GAPI) [9], Analytical Greenness Metric (AGREE) [10], Carbon Footprint Analysis [11], Blue Applicability Grade Index (BAGI) [12], Red–Green–Blue 12 (RGB 12) [13], and Need, Quality, and Sustainability (NQS) [14]. These tools provide quantitative metrics and visual representations that enable researchers to optimize their methodologies according to environmental criteria.

Traditional analytical approaches, particularly chromatographic techniques, often fail to fully align with the concepts of both GAC and WAC due to their reliance on huge amounts of dangerous organic solvents, complex sample preparation protocols, high energy consumption, and the need for sophisticated, affluent equipment [15–17]. Consequently, the development of straightforward, sustainable, and environmentally friendly analytical methods remains a high-priority goal in the field.

Spectroscopic techniques, in contrast, are regarded as an environmentally friendly analytical techniques due to its non-destructive nature, minimal sample preparation requirements, reduced use of chemicals, rapid analysis, and energy efficiency [18, 19]. Among the various spectroscopic techniques, UV spectrophotometry utilizing green solvents stands out as an effective method that fits well with the concepts of both GAC and WAC. Its benefits include simplicity, high selectivity, sensitivity, ease of use, affordability, minimal solvent consumption, stability, reproducibility, environmental sustainability, and its non-destructive characteristics, allowing samples to be reused without the need for pretreatment [20–22].

The analytical capabilities of UV spectrophotometry are significantly enhanced when coupled with chemometric models. This integration creates a powerful analytical platform capable of extracting critical information from complex spectral data, enabling rapid screening, process monitoring, and quality control across diverse applications. Chemometric models such as Classical Least Squares (CLS), Partial Least Squares (PLS), and Multivariate Curve Resolution-Alternating Least Squares (MCR-ALS) facilitate the quantification of multiple components in pharmaceutical formulations despite spectral overlaps and interferences [21, 23].

Despite the advantages, many existing chemometric models depend on randomized data sampling to generate training and validation sets. While this approach is simple, it often results in validation sets that fail to represent the full spectrum of sample variability, potentially resulting in partial model accuracy and undermining reliability and resource efficiency [24, 25]. To handle this matter, the current study introduces the use of D-optimal design created by MATLAB’s Candexch algorithm (MCA)[26]**.** This advanced statistical method constructs genuinely representative validation sets, making certain that validation samples reflect the entire range and distribution of variables across the sample space. This approach enables a thorough and unbiased evaluation of the anticipated performance of chemometric models. Additionally, the D-optimal design contributes to analytical sustainability by minimizing resource usage and waste production while improving reliability through the use of fewer but more representative validation samples.

Motion sickness is a CNS disorder caused by sensory conflicts among visual, vestibular, and proprioceptive inputs. Symptoms include nausea, vertigo, pallor, cold sweats, and autonomic disturbances arise from brainstem vestibular nuclei activation, with the cerebellum coordinating sensory integration. Neuroimaging shows involvement of vestibular cortical and insular areas. Histaminergic and cholinergic pathways contribute, guiding antihistaminergic and anticholinergic treatments[27]. The combination of cinnarizine (CIN) and domperidone (DOM) offers synergistic benefits in motion sickness management.

To demonstrate the effectiveness of our approach, we selected the simultaneous determination of CIN, DOM, and benzophenone (BNZ) as a model system. This combination presents a particularly challenging analytical scenario due to the pharmaceutical importance of CIN and DOM, both individually and in combination formulations, and the critical need to monitor BNZ, a carcinogenic degradation product of CIN.

**Fig. 1:**
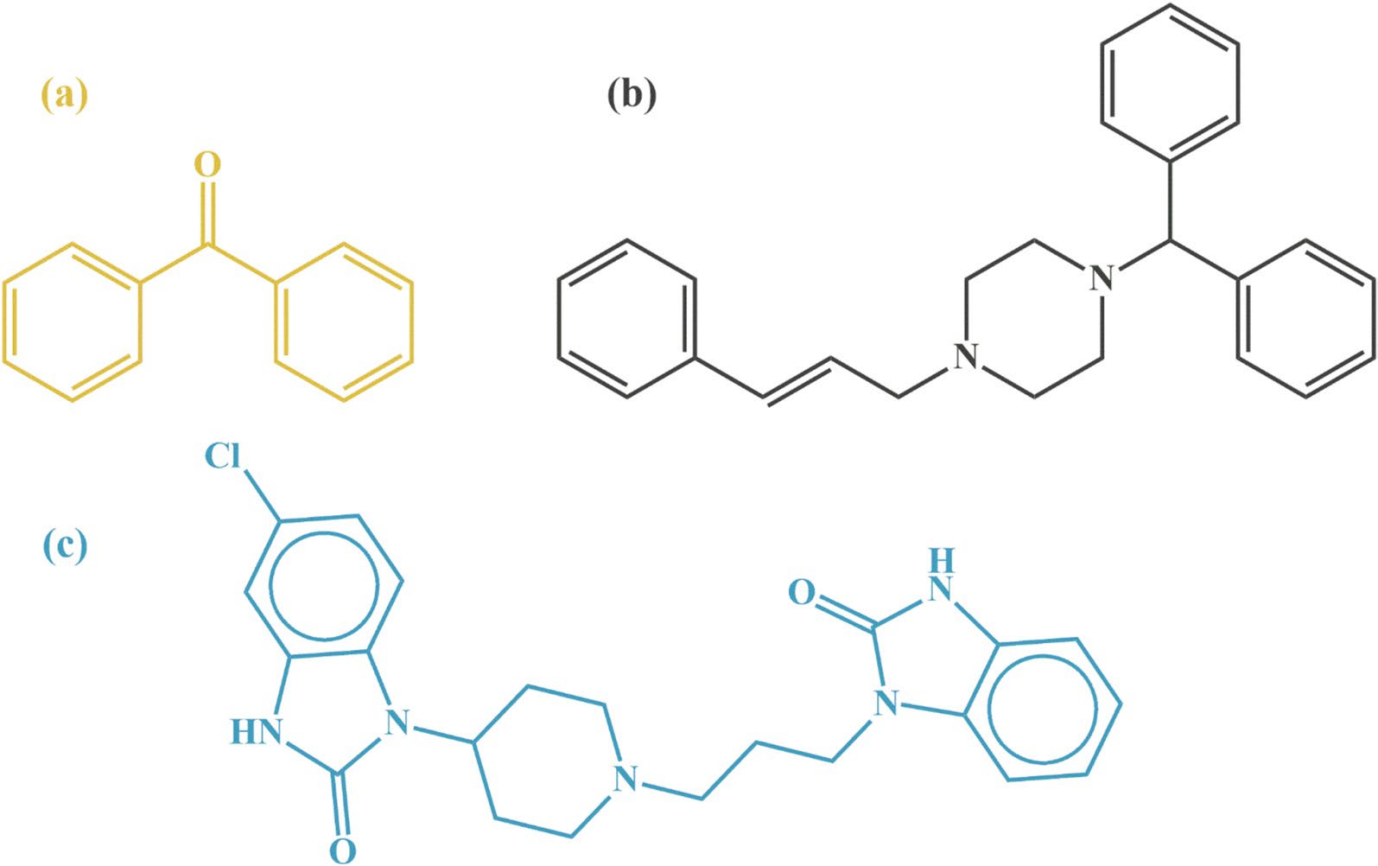
The chemical structure of (**a**) BNZ, (**b**) CIN, and (**c**) DOM

CIN, chemically defined as (E)-1-(diphenyl methyl)-4-(3-phenylprop-2-enyl) piperazine (Fig. 1), is a potent antihistamine commonly used to treat motion sickness [28], which affects nearly 80% of the population to varying degrees [29]. DOM, or 5-chloro-1-[1-[3-(2,3-dihydro-2-oxo-1H-benzimidazol-1-yl) propyl]-4-piperidinyl]-1,3-dihydro-2H-benzimidazol-2-one (Fig. 1), is an antidopaminergic drug frequently used as an antiemetic to prevent nausea and vomiting [28]. BNZ, also known as diphenylmethanone (Fig. 1), is a harmful degradation product of CIN that is carcinogenic and can lead to hepatomegaly [30–32]. Our comprehensive literature review revealed a notable gap: no existing methods have been published for the simultaneous quantification of CIN, DOM, and BNZ.

This study aims to establish a novel analytical framework that integrates sustainability, eco-friendliness, whiteness, and superior analytical performance through the development of UV spectrophotometric methods enhanced by chemometric models and optimized using D-optimal design. The proposed methodology offers a cost-effective, highly sensitive, and environmentally responsible approach for the rapid detection and quantification of CIN, DOM, and BNZ in pharmaceutical formulations. By comprehensively assessing profiles of the greenness and whiteness of our approach utilizing multiple assessment tools and comparing it with reported method [33], we position UV spectrophotometry coupled with advanced chemometric models as a powerful analytical solution for quality control applications, providing a sustainable alternative to conventional, resource-intensive, and environmentally detrimental methods.

## Experimentation

### Reagents and materials

CIN and DOM powders with high purities of 99.1% and 99.6%, respectively, were sourced from the Chemical Industries Development (CID) Company (Giza, Egypt). Additional chemicals, including BNZ (purity 99%) and ethanol HPLC grade (purity ≥ 99.9%) were obtained from Sigma-Aldrich (St. Louis, MO, USA). Vertigun 20/15® tablets, produced by Sigma Company for Pharmaceutical Industries (Menoufia, Egypt) were confirmed to contain 20 mg of CIN and 15 mg of DOM per tablet and were obtained from a community pharmacy.

### Instruments and software

The research utilized a Shimadzu UV-1800 PC double-beam UV–visible spectrophotometer (Shimadzu Corporation, Kyoto, Japan), operated using UVProbe software version 2.42. An electronic Shimadzu balance (Model AY220, Shimadzu Corporation, Kyoto, Japan) was used for weighing, and sample sonication was carried out using a Laboid International LUC-series ultrasonic cleaner (Laboid International, Haryana, India).

MATLAB® R2023b (version 8.2.0.701, MathWorks Inc., Natick, MA, USA) was used to perform chemometric analyses, including CLS, PLS, and MCR-ALS models. The PLS_Toolbox (version 9.0, Eigenvector Research Inc., Manson, WA, USA) and the MCR-ALS GUI 2.0 toolbox, developed by Jaumot, de Juan, and Tauler (Barcelona, Spain), were employed during the modelling process.

### Green solvent selection

#### GSST

**Fig. 2:**
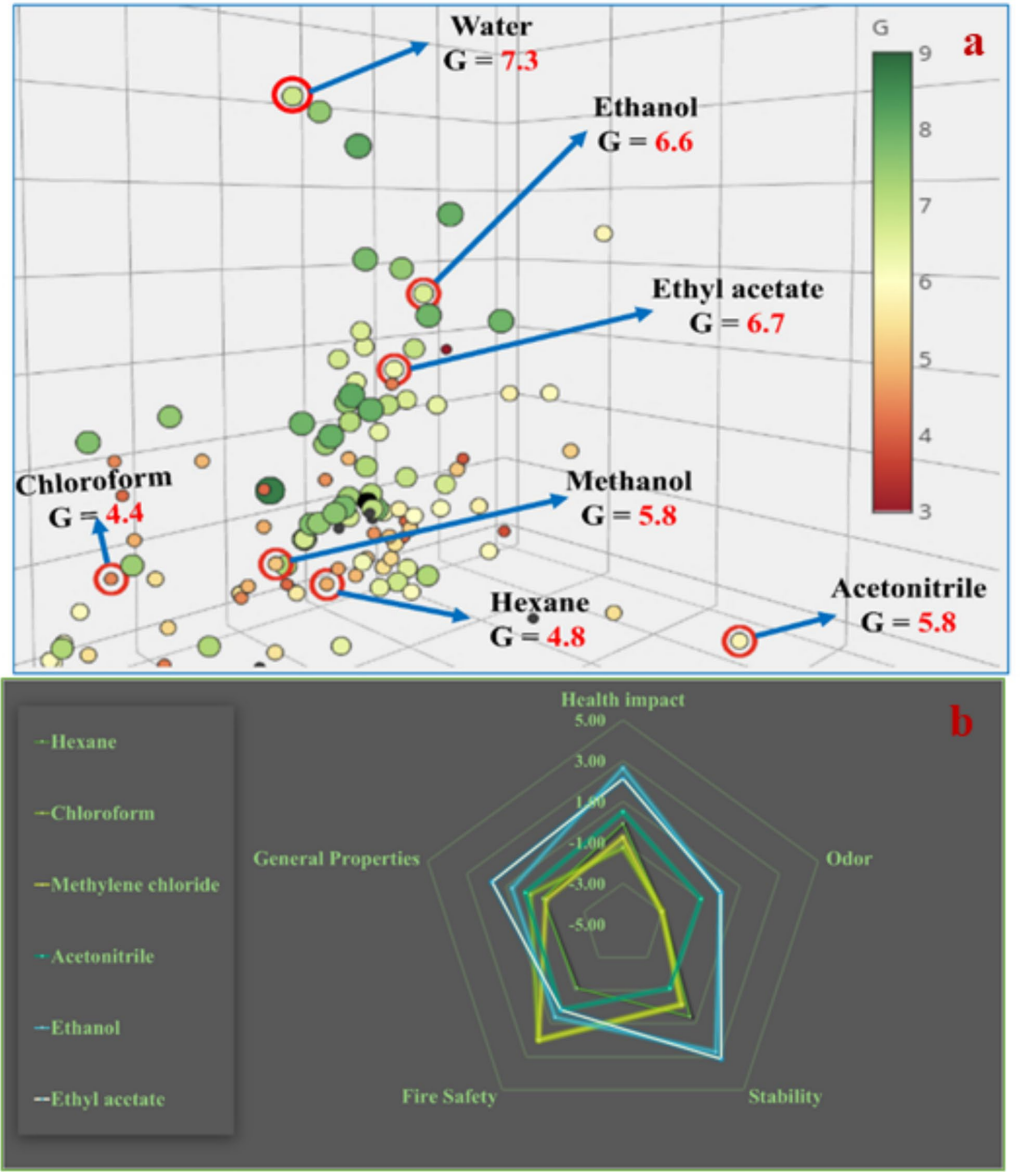
(**a**) G scores by GSST (**b**) Spider chart by SDAGI

Choosing suitable green solvents is essential to achieving sustainability in analytical processes. Well-known pharmaceutical firms, including Pfizer, Novartis, AstraZeneca, and Sanofi, have created solvent sustainability standards that assess and classify solvents based on safety data sheets (SDS), analyzing their merits and drawbacks. To streamline this approach, we employed a newly developed solvent selection tool [34], which allows for the quantitative evaluation and comparison of solvents according to critical environmental, health, safety, and disposal of waste criteria. Each solvent's composite greenness score (G) is determined by this program, where elevated scores signify enhanced sustainability. We utilized this technology to evaluate prospective solvent alternatives and uncover environmentally friendly options to substitute hazardous solvents typically employed in conventional techniques. The software analysis indicated that ethanol had a markedly superior G score relative to harmful solvents such like acetonitrile, methylene chloride, hexane, and chloroform, as illustrated in (Fig. 2a). Ethanol attained elevated G ratings of 6.7 and 6.6, indicating its advantageous performance in disposal of waste, the impact on health, environmental consequences, and safety areas. Conversely, chloroform received a substantially lower score of 4.4, reflecting its inferior greenness scores in these domains. Following these assessments, we selected ethanol as a more environmentally benign solvent, consistent with our objective to establish an analytical method that adheres to green chemistry principles by employing safer, more sustainable chemicals.

#### SDAGI

Although tools such as the GSST are useful for first solvent screening, a more thorough evaluation of the greenness of the reagents, incorporates comprehensive experimental data. The SDAGI tool offers a respected qualitative technique for determining the greenness of solvents and reagents, relying on safety data sheets (SDS). This approach utilizes SDS information to evaluate the attributes of reagents in terms of Safety, Health, and Environment (SHE) impacts. The data is then compiled into greenness scores and displayed through spider diagrams that visually represent the overall greenness of a solvent across key parameters. These scores are categorized into five sections: Stability, Fire Safety, Odor, General Properties, and Health Impact, with score values ranging from − 5 to + 5. Secondary spider diagrams provide additional detail on these subgroups. This visualization approach allows for convenient comparison and assessment of reagents. The % of data utilized to create the Greenness Index is recorded in (Table S1) [35].

In this study, SDAGI analysis, based on SDS data, revealed that ethanol had substantially bigger safety zones and greater average greenness values compared to more hazardous solvents such as chloroform, n-hexane, methylene chloride, and acetonitrile, which are commonly utilized in established methods. When comparing the solvents used in the newly developed method with those in the reported methods, ethanol scored higher with an average score of 1.33, while chloroform, hexane, methylene chloride, and acetonitrile received scores of − 0.06, − 0.91, − 0.12, and − 0.30, respectively, as shown in (Fig. 2b). In comparison to water and ethyl acetate, we observed that ethanol offered a more satisfactory spectral shape and a higher UV sensitivity besides being eco-friendly. This comparison supports the idea that the solvents chosen in the new method offer significant greenness improvements over those previously used, validating the green chemistry principles behind the solvent selection. Consequently, the solvents employed in the developed method are recommended for their environmental benefits, as summarized in (Table S1).

### Standard solutions

Standard working solutions of CIN 100 µg/mL and DOM 75 µg/mL were prepared by dissolving the respective amounts of each compound in ethanol within 250 mL volumetric flasks, followed by dilution to the mark with ethanol. For BNZ, a working solution of 25 µg/mL was prepared by transferring 5 mL of a stock solution (1250 µg/mL) into a 250 mL volumetric flask and diluting it to the mark with ethanol. The solutions were sonicated for 10 min to guarantee thorough dissolution. The calibration and validation datasets were prepared by transferring accurate aliquots from these working standard solutions into 25 mL volumetric flasks and diluted to the specified volume with ethanol.

### Working range and spectral properties

**Fig. 3:**
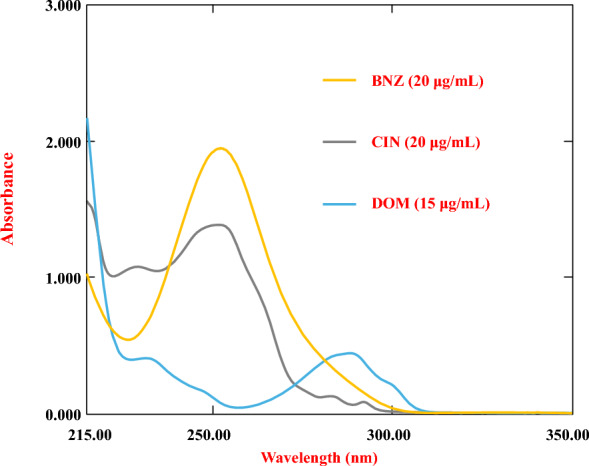
The first-order absorption spectrum of BNZ, CIN, and DOM

To characterize the spectral profiles of the target compounds, Individual UV absorption spectra were obtained. for CIN, DOM, and BNZ across the wavelength range of 215–350 nm (Fig. 3). Standard solutions were prepared at concentrations of 20 µg/mL for CIN, 15 µg/mL for DOM, and 20 µg/mL for BNZ. These specific concentration levels were deliberately selected to provide optimal spectral responses while maintaining relevance to pharmaceutical formulation ratios and ensuring alignment with the method's linear dynamic range.

For the establishment of appropriate working ranges, we conducted systematic spectral analysis of CIN, DOM, and BNZ mixtures by scanning across the 215–350 nm region. Through careful optimization experiments, we determined the ideal concentration working ranges for each analyte: 4–20 µg/mL for CIN, 3–15 µg/mL for DOM, and 1–5 µg/mL for BNZ. These concentration intervals were selected based on multiple criteria: they exhibited linear spectrophotometric responses, provided adequate sensitivity for the detection and quantification of each compound, and corresponded to clinically relevant concentration levels expected in pharmaceutical formulations. This strategic selection of working ranges ensured robust analytical performance across the concentration domains pertinent to quality control applications and therapeutic monitoring.

### Design of experiments

A well-structured experimental framework is critical to guarantee the reliability and relevance of the collected data. The calibration set was constructed using three factors at five levels, resulting in 25 laboratory-prepared drug mixtures with concentration ranges of 4–20 µg/mL for CIN, 3–15 µg/mL for DOM, and 1–5 µg/mL for BNZ, following the Brereton multilevel multifactor approach (Table 1) [36]. Furthermore, an independent validation set involving 13 mixtures was prepared separately. These validation mixtures were strategically selected to cover the concentration range of the model but were not involved in its development. The MCA approach was utilized to ensure the validation set effectively represented the concentration range, providing a robust mechanism for model validation. This independent validation dataset was used to evaluate the model's generalization to unknown data, as indicated in (Table 1). To create the calibration and validation mixtures, suitable aliquots of the standard working solution for each chemical were transferred into 25-mL volumetric flasks and diluted with ethanol to the appropriate level. This rigorous experimental design guaranteed full representation of the concentration ranges, generating a balanced dataset for both calibration and validation. This approach facilitated the effective development and validation of the chemometric models.

**Table 1:** The five-level three-factor experimental design of 25 calibrations mixtures together with the 13 validation set mixtures used in the chemometric methods

Mix no	Calibration set (µg/mL)		
	CIN	DOM	BNZ
1	12	9	3
2	12	3	1
3	4	3	5
4	4	15	2
5	20	6	5
6	8	15	3
7	20	9	2
8	12	6	2
9	8	6	4
10	8	12	5
11	16	15	4
12	20	12	3
13	16	9	5
14	12	15	5
15	20	15	1
16	20	3	4
17	4	12	1
18	16	3	3
19	4	9	4
20	12	12	4
21	16	12	2
22	16	6	1
23	8	3	2
24	4	6	3
25	8	9	1

Mix no	Validation set (µg/mL)		
	CIN	DOM	BNZ
1	13	6	4
2	13	11	3
3	6	5	3
4	14	5	2
5	10	12	4
6	5	10	1
7	8	14	2
8	10	7	1
9	17	3	2
10	18	12	4
11	7	14	4
12	20	8	3
13	15	9	5

### Method development

Our study utilized three different chemometric regression models: CLS, PLS, and MCR-ALS. In chemometric models, chemometricians often begin with CLS because it is simple, rapid, directly based on the Beer–Lambert law and effective when all absorbing analytes are known and the system behaves ideally but CLS struggled with nonlinear interactions and interference, so chemometricians shift to PLS as it extracts latent variables and handles complex, noisy data, enabling accurate quantification even in the presence of spectral overlap and multicollinearity. MCR-ALS is used when deeper resolution is required, especially in complex mixtures with overlapping signals or unknown components. It determined both pure spectral and concentration profiles without requiring prior knowledge. Chemometric best practices emphasize how these models complement one another. CLS and PLS provide fast, practical tools for initial quantification, while MCR-ALS delivers advanced resolution and interpretability when deeper insight is required. A wavelength-specific regression approach was employed for the CLS model, eliminating the need for latent variables (LVs). To boost model effectiveness, a moving window wavelength choice technique was used, systematically testing window widths between 5 and 30 nm via cross-validation. This method helped determine the optimal spectral smoothing level, efficiently reducing noise while maintaining essential quantitative information. In creating the PLS model, we explored various numbers of LVs, ranging from 1 to 10, and selected the best number based on Venetian blinds cross-validation, using the root mean square error of cross-validation (RMSECV) as the performance metric. By using this method, we were able to balance complexity and model fit. For the MCR-ALS model, constraint improvement was crucial in refining the calibration process. Non-negative least squares (NNL) constraints were employed to both spectral profiles and concentration values, leading to improved parameter estimation with fewer iterations and enhanced computational efficiency. This thorough optimization aimed to create dependable and accurate chemometric models for the analysis of complex multi-component pharmaceutical formulations.

Several analytical performing metrics were calculated to judge the final chemometric models [25]. Concerning the calibration dataset, standard error of calibration (SEC), the root mean square error of calibration (RMSEC), and RMSECV were calculated. Regarding the validation set, the root mean square error of prediction (RMSEP) was determined to evaluate the generalizability of the model, while the Relative root mean square error of prediction (RRMSEP) demonstrated how accurately the model performed. Additionally, the bias-corrected mean square error of prediction (BCRMSEP) was calculated to estimate the precision and variations in estimates of newer samples.

The subsequent formula is utilized to calculate the RMSECV, RMSEP, and RMSEC[25]:

$$ RMSE = \sqrt {\frac{{\mathop \sum \nolimits_{i = 1}^{n} (Ei - \hat{E}i)^{2} }}{n}} $$

The other merit figures were determined using the subsequent formulas:

$$ Bias = \frac{{\sum\nolimits_{i = 1}^{n} {\left( {Ei - \hat{E}i} \right)} }}{n} $$

$$ SEC = \sqrt {\frac{{\mathop \sum \nolimits_{i = 1}^{n} (Ei - \hat{E}i - bias )^{2} }}{n - 1}} $$

$$ RRMSEP\left( \% \right) = 100\sqrt {\frac{{\sum\nolimits_{i = 1}^{n} {\left( {Ei - \hat{E}i} \right)^{2} } }}{{\sum\nolimits_{i = 1}^{n} {Ei^{2} } }}} $$

where ${\mathrm{Ei}}$ represents the actual analyte concentration in sample i, $\hat{E}i$ is the predicted concentration, and n is the total number of samples in the validation set.

A number of other parameters were also calculated, such as robustness, accuracy, precision, and the limits of quantification (LOQ) and detection (LOD) [37]. Using triplicate measurements, the percentage recovery (%R) at different concentration levels for all analytes were calculated in order to assess accuracy for CIN at [8, 12, and 16 µg/mL], DOM at [6, 9, and 12 µg/mL], and BNZ at [2, 3, and 4 µg/mL]. The relative standard deviation (%RSD) was used to evaluate precision, considering both intra-day and inter-day evaluations. Three different samples were determined in triplicate at concentration of [8, 12, and 16 µg/mL] for CIN, [6, 9, and 12 µg/mL] for DOM, and [2, 3, and 4 µg/mL] for BNZ, with intra-day tests conducted on the same day and inter-day tests conducted across three separate days. The method's robustness was tested by varying specific parameters while keeping others constant. Three variables were selected: slight changes in intervals of wavelength (1.1 nm versus 1 nm), the speed of scan (medium versus fast), and spectral bandwidths (0.9 nm versus 1 nm slit width). The robustness was demonstrated by its ability to maintain reliable results despite these minor variations. Finally, the LOD and LOQ were calculated utilizing the net analyte signal method [21].

### Pharmaceutical dosage application

Ten tablets were weighed, ground into a fine powder, and mixed thoroughly. A specific quantity of powder, equivalent to 20 mg of CIN and 15 mg of DOM, was meticulously weighed in a 100-mL volumetric flask using ethanol as a dissolving agent and subjected to ultrasonication for 10 min. Ethanol was then added to get the volume up to the mark. After filtering, 0.5 mL of the filtrate was transferred into a 10 mL measuring flask. The leftover volume was subsequently completed with ethanol to the designated mark, yielding final concentrations of 10 and 7.5 μg/mL for CIN and DOM, respectively. Ethanol served as the blank for recording the absorption spectra.

## Results and discussion

### Method development

The complex spectral profiles of CIN, DOM, and BNZ show substantial overlapping in their spectra of UV absorption as shown in (Fig. 3). By using the whole spectrum data from the three-component mixture, we created and refined three multivariate chemometric models to tackle this analytical concern. Wavelengths above 300 nm were omitted due to the low absorbance values in this range. Additionally, wavelengths below 220 nm were removed to minimize interference from noisy spectra. Therefore, the chemometric models, CLS, PLS, and MCR-ALS, utilized wavelengths between 220 and 300 nm, with a 1 nm interval, leading to 81 data points per spectrum. These data points functioned as input for the creation and optimization of the models, which were implemented using MATLAB® software.

### Calibration set design

To develop the calibration set, a multilevel and multifactor experimental approach was employed to optimize the combination of the three components, resulting in 25 distinct mixtures, following Brereton et al.'s recommendations [36]. This approach significantly enhances model accuracy by generating uncorrelated analyte concentration profiles. Five concentration levels were tested (− 2, − 1, 0, + 1, + 2), with 0 as the midpoint, ensuring that variations in concentrations minimized correlation between analyte profiles, as shown in (Table 1). This permitted the model to distinguish the spectral contribution of each component more effectively. By structuring the variation in this way, we improved quantification accuracy and prevented overfitting. Furthermore, this design reduced laboratory resource consumption, minimizing material waste, chemical waste, and calibration sample requirements. Ultimately, the multilevel, multifactor approach improved model reliability and accuracy while adhering to environmentally sustainable principles in analytical method development.

### Validation set design

**Fig. 4:**
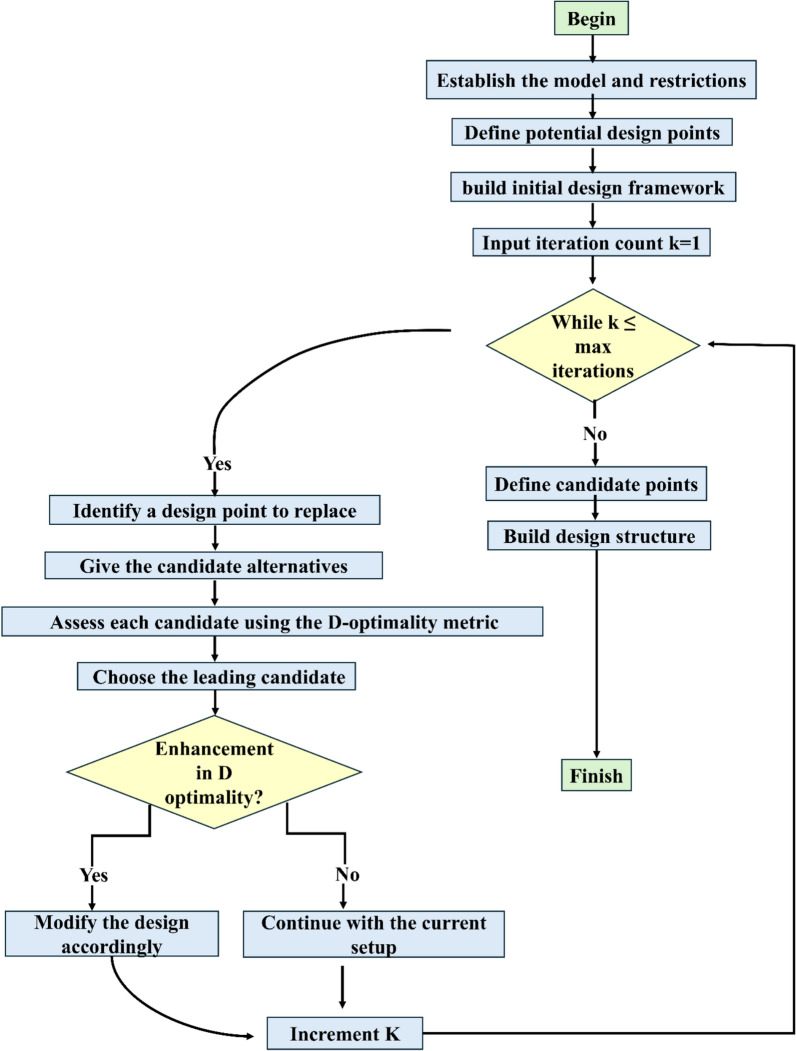
Flowchart of MCA steps for D-optimal design

**Fig. 5:**
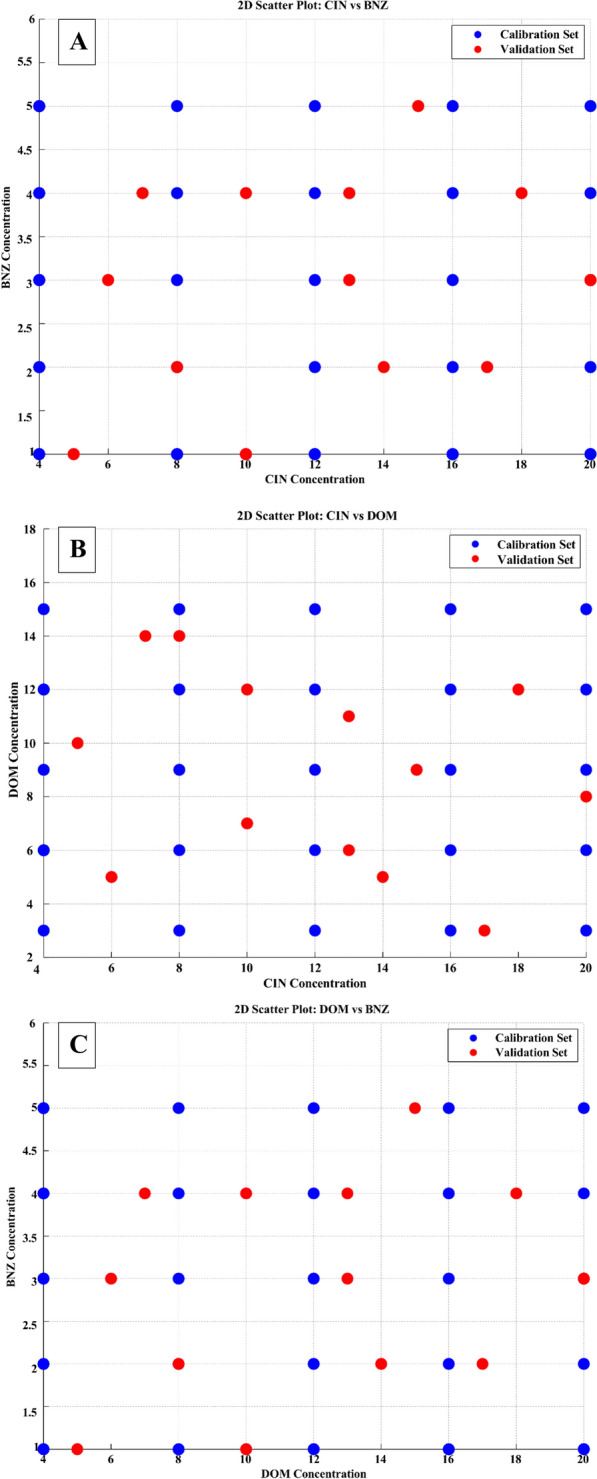
2D scatter plot of the validation set of (**A**) CIN versus BNZ, (**B**) CIN versus DOM, and (**C**) DOM versus BNZ designed by MCA design as optimal-space filling design

**Fig. 6:**
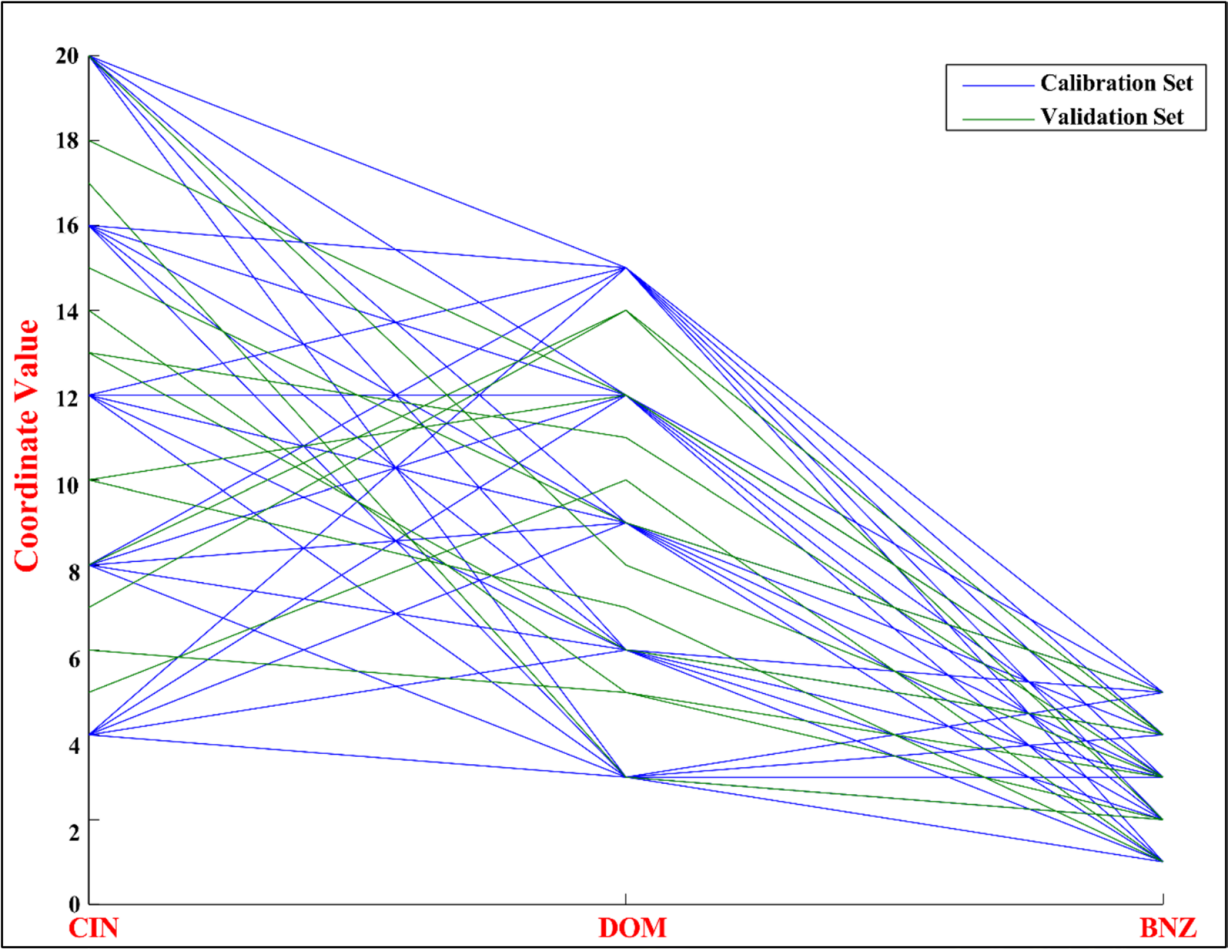
Parallel coordinate plot of analytes concentrations in validation and calibration sets

When creating chemometric models for the analysis of multi-component pharmaceuticals, building a strong and representative validation set is crucial to guarantee the model's reliability and predictive precision. To address the shortcomings of basic random sampling methods, which frequently struggle to cover the full concentration range of analytes effectively, we utilized a D-optimal design developed through MCA for constructing the validation set. This advanced statistical method provides significant benefits in terms of thorough coverage of the design space, exacting model evaluation, and improved analytical effectiveness. The MCA utilizes an iterative optimization process to maximize the determinant of the information matrix (|X'X|), thereby minimizing the variance of parameter estimates in the regression model. This optimization produces an experimental design that effectively captures the complex interactions within multicomponent systems. (Fig. 4) provides a comprehensive flow chart that illustrates the iterative nature of the algorithm and crucial steps in decision-making in creating the D-optimal design. Using MATLAB R2023b , custom scripts were developed to implement the algorithm, enabling the systematic selection of the candidate's highest informative samples. This carefully structured approach ensured broad coverage of the multidimensional analyte space, yielding a compact yet highly informative 13-mixture validation set across the entire analyte range, as shown in the scatter plots (Fig. 5). This set demonstrates exceptional capability in assessing the performance of chemometric models to predict across the full spectrum of sample components (Table 1). The effectiveness of the MCA-generated validation set is visually depicted in a parallel coordinate plot (Fig. 6), which provides a comprehensive visualization of the concentration distribution patterns across the three target analytes (CIN, DOM, and BNZ) in both calibration (n = 25, green lines) and validation (n = 13, blue lines) sample sets. Several critical insights emerge from this visualization. First, the validation set (blue trajectories) exhibits deliberate coverage of the entire concentration domain for each analyte, spanning from lower boundary values (4 μg/mL for CIN, 3 μg/mL for DOM, and 1 μg/mL for BNZ) to upper boundary values (20 μg/mL for CIN, 15 μg/mL for DOM, and 5 μg/mL for BNZ). This systematic distribution ensures that the model's performance is evaluated across the full analytical working range for each compound. More importantly, the plot reveals the sophisticated interrelationships between analyte concentrations. The complex crossing patterns of the trajectory lines between adjacent analyte axes illustrate the diverse concentration combinations present in both calibration and validation sets. This complexity is particularly noteworthy in the transitions between CIN-DOM and DOM-BNZ, where numerous intersecting trajectories indicate comprehensive coverage of potential concentration ratios and combinations. The extensive overlap between calibration (green) and validation (blue) trajectories confirms that the validation set effectively captures the multidimensional concentration space defined by the calibration samples. Notably, the D-optimal design has strategically positioned validation samples to represent both boundary conditions and intermediate concentration values, creating an optimal evaluation framework with only 13 samples, a significant efficiency improvement over conventional approaches requiring larger validation sets. The plot demonstrates that our analytical method has been validated against a statistically optimized subset of samples that thoroughly represents the potential concentration combinations encountered in real-world pharmaceutical formulations and stability studies, thereby enhancing the reliability and robustness of the developed chemometric models. Overall, the strategic use of the D-optimal design offers several significant benefits over traditional random sampling methods. This incorporated greater predictive reliability enhanced the sustainability of methods, robust model assessment, and optimized resource use. This design minimizes the possibility of biased accuracy determination and streamlines the validation process by providing even and consistent coverage of the design area. With respect to this efficiency, less material is used, and less waste is produced without sacrificing analytical quality. Additionally, Multivariate systems benefit greatly from the D-optimal design's capacity to optimize the design space while taking variable correlations into consideration as a result, the validation set exhibits increased sensitivity to interactions between analytes and comprehensively covers the entire concentration range, a critical consideration for complex pharmaceutical dosage forms.

In summary, the use of the MCA-generated D-optimal design for creating validation sets marks a notable step forward in chemometric model validation. This design ensures the development of reliable, robust, and ecologically friendly analytical methods by establishing ideal balance between comprehensive design space coverage and analytical sustainability. We believe that implementing this advanced design approach could reduce development time and expenses in pharmaceutical quality control, which would be in line with industry’s increasing demand for fast, accurate, and eco-friendly methods for analysis.

### CLS

The CLS model, which relies on multivariate linear regression depending on the Beer-Lambert law, was initially implemented using the K-matrix calibration method [38]. This approach assumes that absorbance and concentration are linearly related for each component. Although the CLS model was initially built using the calibration set, its performance was suboptimal. However, by introducing an intercept term into the regression, the model's predictions improved significantly, which resulted in excellent recovery percentages of 99.52, 98.96, and 99.28% for CIN, DOM, and BNZ, respectively, (Table 2). Despite its simplicity, CLS struggled with nonlinear interactions and interference. While it provided a reasonable foundation, it was eventually surpassed by more advanced chemometric models in terms of performance.

**Table 2:** Validation sheet and regression parameters by the proposed methods

Parameters	CLS			PLS			MCR-ALS		
	BNZ	DOM	CIN	BNZ	DOM	CIN	BNZ	DOM	CIN
Range (μg/mL)	1–5	3–15	4–20	1–5	3–15	4–20	1–5	3–15	4–20
Slope ^a^	0.982	0.987	1.023	1.011	0.990	1.015	0.998	0.995	1.001
Intercept ^a^	0.001	0.0005	0.0008	0.001	0.0003	0.0006	0.0008	0.0001	0.0004
r^2 a^	0.9993	0.9995	0.9994	0.9995	0.9997	0.9996	0.9997	0.9999	0.9998
Mean for calibration set	99.280	98.960	99.520	99.780	99.980	99.690	100.120	100.010	99.970
Mean for validation set	100.320	99.960	100.520	100.430	99.830	100.610	100.620	100.190	100.520
SD of calibration set	1.923	0.923	0.629	1.6189	0.406	0.145	0.986	0.224	0.092
SD of validation set	1.811	1.6235	1.856	1.802	1.522	1.749	1.193	0.927	1.236
LOD (μg/mL) ^b^	0.254	0.863	0.689	0.230	0.690	0.626	0.209	0.604	0.569
LOQ (μg/mL) ^b^	0.770	2.615	2.089	0.698	2.092	1.897	0.635	1.830	1.725
RMSEC ^c^	0.062	0.042	0.036	0.024	0.020	0.013	0.012	0.009	0.011
RMSEP ^d^	0.042	0.127	0.201	0.035	0.097	0.187	0.019	0.022	0.154
RRMSEP ^e^	3.781	2.345	3.017	3.433	2.129	2.737	3.121	1.936	2.487
BCRMSEP ^f^	0.089	0.471	0.240	0.081	0.428	0.218	0.074	0.389	0.198
SEC ^g^	0.085	0.449	0.227	0.077	0.408	0.206	0.070	0.371	0.187
Accuracy ^h^ (M.R % ± S.D)	99.73 ± 0.782	100.24 ± 0.893	99.86 ± 0.821	99.88 ± 0.710	100.13 ± 0.812	99.97 ± 0.745	99.99 ± 0.646	100.05 ± 0.739	100.06 ± 0.677
Repeatability ^h^ (%RSD)	0.623	0.745	0.687	0.565	0.677	0.623	0.514	0.616	0.566
Intermediate precision ^h^ (%RSD)	0.687	0.812	0.753	0.623	0.737	0.683	0.566	0.671	0.621
Robustness ^h^ (%RSD)	0.928	0.653	0.891	0.851	0.878	0.911	0.593	0.707	0.652
Experimental t value ^i^	0.839	1.568	2.359	1.294	2.349	0.174	0.959	1.854	0.598
Critical value t_(0.05, 12)_	2.179	2.179	2.179	2.179	2.179	2.179	2.179	2.179	2.179
Critical value t_(0.01, 12)_	3.055	3.055	3.055	3.055	3.055	3.055	3.055	3.055	3.055

^a^Data of the straight line plotted between predicted concentrations versus actual concentrations of the calibration set

^b^The LOD and LOQ calculations are based on the net analyte signals

^c^Root means square error of calibration

^d^Root means square error of prediction

^e^Relative root means square error of prediction

^f^Bias corrected mean square error of prediction

^g^standard error of calibration

^h^Average of three determinations

^i^t_exp_ =|100 − R̄_exp_|× √n/S_R_, where: R̄exp is the mean recovery percentage, n is the number of samples (13), and S_R_ is the standard deviation of recoveries

### PLS model

**Fig. 7:**
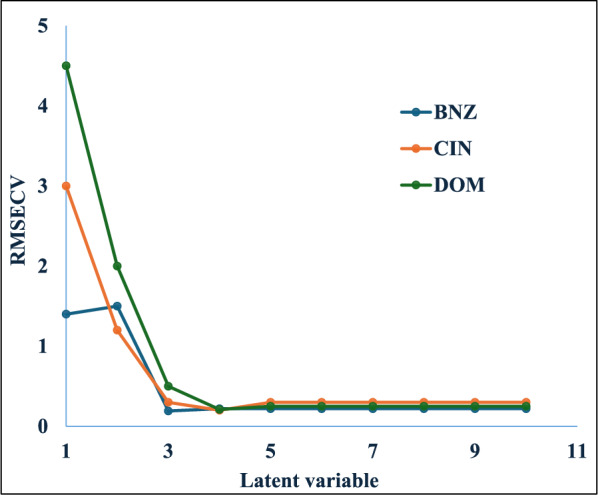
RMSECV plot of the calibration set as a function of the optimum LVs for the PLS model

PLS, one of the most extensively used chemometric models for multivariate calibration, was employed using MATLAB® and PLS Toolbox 9.0. Cross-validation, where one sample is excluded at a time, was applied to optimize the model [39]. The optimal number of LVs was determined following the established criteria of Haaland and Thomas[40], which balances model complexity with predictive capability. Four LVs were identified as optimal based on the minimum RMSECV, as illustrated in (Fig. 7). This selection effectively prevented both overfitting and underfitting of the model. The four-component PLS model demonstrated superior modelling performance for all analytes, yielding RMSECV values of 0.201, 0.211, and 0.192 for CIN, DOM, and BNZ, respectively. These values confirm the model's excellent predictive capability while maintaining computational efficiency.

### MCR-ALS model

The MCR-ALS model represents a sophisticated chemometric model for resolving complex multicomponent systems without prior knowledge of individual component characteristics. This technique employs a bilinear decomposition model (D = CS^T + E) to resolve the spectral data matrix D into the product of concentration profiles C and pure spectral profiles S^T, with E representing the residual error matrix. Unlike traditional multivariate methods, MCR-ALS can effectively extract meaningful chemical information even in the presence of spectral overlaps and matrix effects [41, 42].

In our implementation, the MCR-ALS algorithm proceeded through numerous critical steps. First, we applied Evolving Factor Analysis (EFA) for initial estimation of the number of components and their concentration windows, using a log eigenvalue threshold of -4 to distinguish between significant chemical components and noise. This initial estimate confirmed the presence of three distinct spectral components corresponding to CIN, DOM, and BNZ. Subsequently, the ALS optimization procedure was initiated using the EFA results as initial estimates. The optimization iteratively refined both concentration and spectral profiles while applying appropriate constraints to ensure chemically meaningful solutions. The constraints implemented in our model included: non-negativity for both spectral profiles and concentration reflecting their physical–chemical significance, closure constraints for concentration profiles to account for the mass balance, Unimodality for spectral profiles to ensure peak shape consistency, and selectivity constraints in spectral regions where the presence of specific analytes was known. The ALS algorithm reached convergence after 15 attempts, satisfying our established criterion for convergence of less than 0.20% relative change in the standard deviation of residuals between consecutive iterations. The final MCR-ALS model achieved exceptional fit with an explained variance of 99.92% and a lack of fit of 0.0092%, indicating excellent resolution of the three-component system. The recovered spectral profiles showed a strong correlation with the reference spectra (r > 0.998 for all components), confirming the chemical identity of the resolved components.

**Fig. 8:**
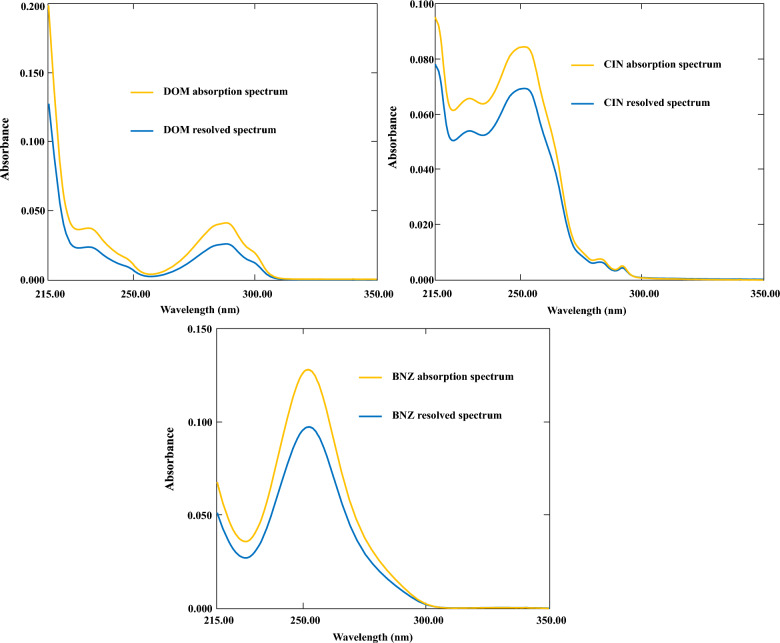
Absorption spectra and resolved spectra estimated by MCR-ALS algorithm

Cosine Similarity Analysis was performed to evaluate the spectral recovery's accuracy by comparing the spectra resolved using MCR-ALS with the pure component spectra that were measured independently. Cosine similarity offers a numerical measure of the degree of alignment between the recovered and true spectra, with values closer to 1 revealing an almost ideal match. The formula for cosine similarity is:

$$ \cos \left( \theta \right) = \left( {A \, \cdot \, B} \right)/\left( {\left| {\left| A \right|} \right| \, ||B||} \right) $$

where A and B represent the recovered and standard spectra, respectively. The analysis demonstrated that the spectral recovery was highly accurate, with cosine similarity values of 0.992, 0.988, and 0.994 for DOM, CIN, and BNZ, respectively, as presented in (Table S2). These values confirm the excellent recovery capability of the MCR-ALS algorithm despite the significant spectral overlap. As shown in (Fig. 8), the resolved spectra (blue lines) closely follow the profile of the reference absorption spectra (yellow lines) for all three analytes, with only minor deviations in peak intensity. For DOM, the characteristic absorption maximum at approximately 285 nm was accurately recovered, while the spectral features in the 225–235 nm range showed slight differences in intensity but maintained the correct profile shape. Similarly, the CIN resolved spectrum successfully captured the dual-peak feature between 240–260 nm, with the characteristic maximum at approximately 250 nm accurately represented. The BNZ spectrum, featuring a prominent absorption band centered at approximately 250 nm, was recovered with the highest fidelity among the three compounds, as indicated by its superior cosine similarity value. The minor intensity differences observed between the resolved and reference spectra can be attributed to residual interactions within the multicomponent system that are inherently challenging to completely eliminate. However, these differences did not significantly impact the quantitative performance of the method, as evidenced by the excellent prediction results reported in the validation studies.

To thoroughly evaluate the reliability of our MCR-ALS model, we conducted a detailed Rotational Ambiguity determination utilizing the MCR-BANDS algorithm (Table S3). This assessment was crucial for quantifying the potential uncertainties in the resolved spectral profiles and their impact on the quantitative determination of CIN, DOM, and BNZ. The analysis focused on establishing the Area of Feasible Solutions (AFS), the Maximum Range of Feasible Solutions (Max RFS), the Average Range of Feasible Solutions (Avg RFS), and identifying essential wavelengths where ambiguity was most prominent. As displayed in (Fig. 8), the resolved spectra anticipated by the MCR-ALS algorithm closely align with the measured absorption spectra for all three analytes, yet certain regions display minor deviations that warrant further investigation through ambiguity analysis. DOM exhibited an AFS of 5.8%, with an average RFS of 0.009 AU and a maximum RFS of 0.021 AU occurring at a critical wavelength of 285 ± 2 nm. This resulted in a little effect on the quantification (< 1.7%). The selectivity index for DOM was calculated as 0.92, indicating excellent resolution despite the moderate ambiguity, primarily due to its distinct absorption maximum of around 280 nm with minimal spectral overlap with the other components. CIN demonstrated the highest ambiguity among the three analytes, with an AFS of 8.2%, an average RFS of 0.014 AU, and a maximum RFS of 0.027 AU at a crucial wavelength of 253 ± 2 nm, leading to a moderate impact on quantitation (< 2.9%). This higher ambiguity can be attributed to the spectral overlap with BNZ, particularly in the 240–260 nm region as evident in (Fig. 8). Despite this challenge, CIN maintained a selectivity index of 0.87, which remained sufficient for reliable quantitation. BNZ showed intermediate ambiguity with an AFS of 6.5%, an average RFS of 0.011 AU, and a maximum RFS of 0.023 AU at 252 ± 2 nm, leading to a slight effect on quantitation (< 2.1%). The selectivity index for BNZ was 0.89, indicating good resolution. The prominent absorption maximum of BNZ around 250 nm, clearly visible in (Fig. 8), contributed significantly to its successful resolution despite some overlap with CIN in this region. With a standard deviation of 1.25% and a total mean AFS of 6.83%, the components showed moderate and constant ambiguity over all components. Rotational ambiguity and its impact on quantitative outcomes are strongly correlated, as seen by the 0.94 correlation coefficient between AFS and quantitation effects. We implemented several constraint strategies to lessen the impact of ambiguity, particularly for CIN where it was most pronounced. Non-negativity constraints were employed for both concentration and spectral profiles, providing fundamental chemical meaning to the solutions. Additionally, correlation constraints based on reference spectra reduced the AFS by an average of 39% across all components. For CIN specifically, the implementation of unimodality constraints further reduced ambiguity by constraining the resolved spectrum to maintain a single peak in the 245–255 nm region. Local rank analysis was also performed at different wavelength regions, revealing that the 230–260 nm region contributed most significantly to the resolution of BNZ, while the 270–290 nm region was critical for DOM determination. For CIN, the entire spectral range was necessary for optimal resolution due to its complex spectral features and overlap with the other components. The constraint combination strategy resulted in final AFS values of 3.5%, 2.2%, and 2.8% for CIN, DOM, and BNZ, respectively, all well below the 5% threshold typically considered acceptable for reliable quantitative analysis. The constrained model exhibited explained variance exceeding 99.9% and lack of fit below 0.1%, demonstrating excellent agreement between the experimental data and the model. This comprehensive ambiguity assessment confirms the robustness of our MCR-ALS model for the simultaneous determination of CIN, DOM, and BNZ, with uncertainties that are well within acceptable limits for analytical purposes. The careful consideration of ambiguity characteristics for each component has been incorporated into the uncertainty calculations for the final concentration determinations, ensuring analytical reliability and integrity.

This comprehensive assessment confirms the superior performance and stability of MCR-ALS compared to CLS and PLS (Table 2). By combining quantitative accuracy with qualitative information, MCR-ALS demonstrated exceptional utility in resolving this complex pharmaceutical formulation where overlapping spectra pose significant analytical challenges.

### Models’ validation

The evaluation of predictive capabilities was carried out using an external validation set that included 13 D optimal design-produced mixtures. These validation combinations were not involved in the model-creating process, but the levels of concentration were still within the working range used to create the models, as shown in (Table 1). To evaluate their anticipated power, the recently built predictive models were employed for these validation combinations. The comparative analysis of CLS, PLS, and MCR-ALS as shown in (Table 2) provides subtle but meaningful variations in calibration performance, sensitivity, and model validation A slope value close to 1 and an intercept value close to 0 indicate improved model accuracy and less bias. Subtle improvement becomes obvious as one progresses from CLS to MCR-ALS. These findings are in line with chemometric literature that shows how well MCR ALS resolves overlapping spectrum and extracts pure component signals, leading to quantitative models that are more accurate.

Furthermore, consistency in model performance is evidenced by the mean recoveries in both calibration and validation sets reinforcing each model’s overall accuracy. Although all three chemometric models deliver excellent linear calibration as reflected by their high correlation coefficients (r^2^ > 0.999), MCR-ALS slightly outperforms both CLS and PLS because of superior slope fidelity and bias minimization conferred by its ability to deconvolute complex spectral overlap and recover pure analyte profiles. The standard deviation (SD) values for the calibration set reveal a progressive tightening of precision from CLS to PLS and ultimately MCR-ALS. Specifically, the SD for CIN drops significantly from 0.629 (CLS) to 0.145 (PLS) and finally to 0.092 (MCR ALS). DOM and BNZ show similar improvements. The superior ability of MCR ALS to separate overlapping spectral features owing to its algorithmic deconvolution which yields substantially reduced noise and enhanced repeatability. The validation sets, while CLS and PLS exhibit somewhat elevated SDs due to extrapolation to unseen mixtures, MCR-ALS continues to outperform, with validation SDs notably lower (e.g., CIN: 1.856 → 1.749 → 1.236). This indicates that MCR-ALS not only fits calibration data more tightly but also generalizes better to novel samples, reaffirming its ability in predictive applications.

In terms of sensitivity, MCR-ALS consistently yields lower LOQ and LOD. The LOD for BNZ decreases from 0.254 (CLS) to 0.209 µg/mL, and the LOQ decreases from 0.770 to 0.635 µg/mL; DOM and CIN also show comparable decreases. These improvements are a reflection of MCR- ALS's superior capacity to resolve weaker signals in complex spectral domains and demonstrate that MCR-ALS enables more reliable quantitation at lower analyte concentrations. This capability is particularly noteworthy in quality control contexts where trace detection and accurate quantification are essential.

Precision was evaluated using intra-day, inter-day, and robustness measurements, all of which were expressed as relative standard deviation (%RSD), for each of the three chemometric models: CLS, PLS, and MCR ALS. Both CLS and PLS are consistently outperformed by MCR ALS, which provides lower %RSD values for all analytes (BNZ, DOM, and CIN), indicating greater precision and stability to small operational disturbances. From CLS to PLS and then to MCR ALS, calibration performance significantly improves, as evidenced by the RMSEC values for BNZ dropping from 0.062 to 0.024 to 0.012 (with comparable trends for DOM and CIN) This indicates progressively tighter model fitting, with MCR-ALS achieving virtually minimal calibration error by utilizing its spectral deconvolution capabilities. Similarly, prediction accuracy on external samples, captured by **RMSEP**, also improves—especially for DOM, which drops from 0.127 (CLS) to 0.097 (PLS) and 0.022 (MCR-ALS). These reductions demonstrate MCR-ALS's superior capacity to generalize to unseen mixtures.The RRMSEP confirms this enhancement in proportion to concentration ranges: CLS yields values up to ~ 3.78%, while MCR-ALS reduces these to as low as ~ 1.94%, indicating markedly better relative accuracy—a critical advantage in trace-level pharmaceutical analysis. The BCRMSEP decreases from 0.089 to 0.074 for BNZ, and similarly for other analytes, confirming that MCR-ALS not only minimizes raw error but also reduces systematic bias in predictions.

Finally, For BNZ and DOM under CLS (t_exp_ = 0.839 and 1.568, respectively) and BNZ and CIN under PLS (1.294 and 0.174), the calculated t-values fall below the 5% (t = 2.179) and 1% (t = 3.055) significance thresholds, indicating no significant bias—except for CIN in CLS and DOM in PLS. In these cases, t_exp_ values (2.359 and 2.349) exceed the 5% threshold but remain below the 1%, suggesting mild yet systematic errors, likely due to unresolved spectral overlap.

In contrast, MCR-ALS yields t_exp_ values well below both thresholds for all analytes (BNZ = 0.959, DOM = 1.854, CIN = 0.598), confirming its robust and unbiased performance even under stringent statistical scrutiny. It is the only method that passes both the 5% and 1% significance tests across all compounds, highlighting its superior capability in resolving complex mixtures. This observation aligns with literature reports emphasizing MCR-ALS’s effectiveness in handling overlapping spectral data.

### The elliptical joint confidence region (EJCR)

**Fig. 9:**
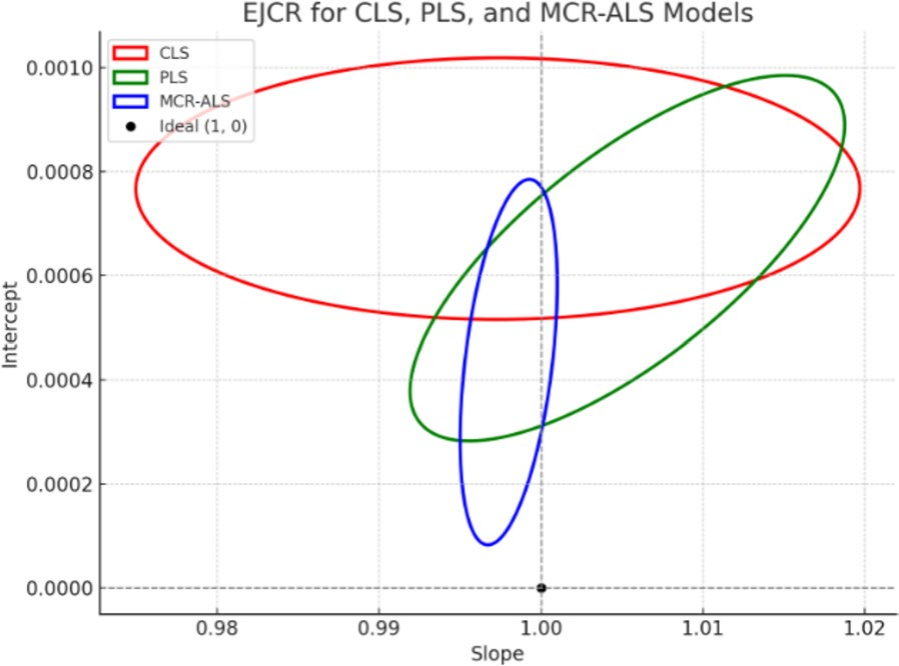
EJCR for chemometric Models in the slope-intercept plane, showing that MCR-ALS was the most efficient Chemometric model

EJCR **is** a graphical and statistical approach used to evaluate the performance of regression models by assessing the variability and uncertainty in both the slope and the intercept. Instead of analysing these two parameters separately, the EJCR method considers them simultaneously [43]. In multivariate calibration—particularly in chemometric modelling such as CLS, PLS, or MCR-ALS—one often seeks to evaluate how close a model’s estimated regression line is to the ideal case, which would have a slope of 1 and an intercept of 0. The ideal represents complete agreement between predicted and actual values, meaning the model is both impartial and proportional. The EJCR captures this relationship by constructing an ellipse around the mean point of the slope and intercept values obtained from multiple model iterations.The ellipse is derived from the covariance of the slope and intercept estimates, and it defines a region that, with a given confidence level (commonly 95%), is expected to contain the true parameter values. The size and position of the ellipse indicate the precision and correlation between the slope and intercept. A tight ellipse centred near the ideal point (1,0) indicates a model with low bias and high precision. Conversely, a larger ellipse, or one that is shifted away from the ideal point, suggests greater uncertainty or potential systematic error. Thus, EJCR is a useful and understandable tool for visually comparing different models. It allows researchers to determine not only how accurate each model is but also how consistent and statistically reliable its predictions are, providing more insight than single-point metrics like RMSEP or correlation coefficients alone. As revealed in Fig. 9, MCR-ALS shows the best performance, with its ellipse tightly clustered near the ideal point (slope ≈ 1, intercept ≈ 0), indicating high accuracy, minimal bias, and excellent precision.PLS also performs well, with an ellipse close to the ideal point but slightly more variable than MCR-ALS, suggesting moderate precision and low bias.CLS performs the weakest, with an ellipse further from the ideal point, reflecting higher bias, and greater variability.

### Chemometric workflow optimization

In chemometric models, analysts often begin with CLS because it is simple, rapid, directly based on the Beer–Lambert law and effective when all absorbing analytes are known and the system behaves ideally but CLS struggled with nonlinear interactions and interference, so analysts shift to PLS as it extracts latent variables and handles complex, noisy data, enabling accurate quantification even in the presence of spectral overlap and multicollinearity. MCR-ALS is used when deeper resolution is required, especially in complex mixtures with overlapping signals or unknown components. It determined both pure spectral and concentration profiles without requiring prior knowledge. Chemometric best practices emphasize how these methods complement one another. CLS and PLS provide fast, practical tools for initial quantification, while MCR-ALS delivers advanced resolution and interpretability when deeper insight is required.

### Statistical analysis

Analysis of variance (ANOVA) revealed no appreciable variation in accuracy between the models that were proposed and the reported method [28] on pharmaceutical formulation.The calculated F-values were less than the referenced F-value, as indicated by the p-values exceeding 0.050, suggesting that there were no statistically significant differences in these models' accuracy in determining CIN and DOM (Table S4).

### Pharmaceutical applications

Vertigun® pills were successfully analyzed using the suggested method, which was unaffected by the additions. The standard addition procedure was used to validate the models, and (Table 3) shows the results.

**Table 3:** Determination of CIN and DOM, in pharmaceutical preparation by the suggested chemometric models and application of standard addition technique

Preparation		%Recovery ± %RSD ^(a)^		
		CLS	PLS	MCR-ALS
CIN	Application	101.990 ± 0.073	101.930 ± 0.046	101.910 ± 0.0261
	Standard addition	100.690 ± 0.825	100.860 ± 0.603	100.980 ± 0.381
DOM	Application	98.890 ± 1.157	99.070 ± 0.913	99.180 ± 0.712
	Standard addition	98.430 ± 0.926	99.310 ± 0.704	99.840 ± 0.482

^a^Average of three determinations

## Evaluation of sustainability of the method

### Greenness evaluation using NEMI

The NEMI provides important insights into the ecological impact of processes and is an initial qualitative tool for assessing their environmental sustainability. Four quadrants are used in a circular figure: waste, corrosive, hazardous, persistent, bioaccumulative, and toxic (PBT). (Fig S1). If the method meets the greenness criteria, the circle is colored green. According to the EPA's Toxic Release Inventory (TRI), these standards ensure that the chemicals in question are not classified as Persistent, Bioaccumulative, or Toxic (PBT). The waste generated shouldn't be more than 50 g, and the pH level should stay between 2 and 12—a non-corrosive range. [44]. We implemented NEMI pictograms that clearly indicated the sustainability of our approaches for the environment. The diagram's four quadrants were filled with green, demonstrating that the method met the necessary greenness requirements conversely to the reported method [33] as illustrated in (Table 4).

**Table 4:** Sustainability assessments of the proposed methods compared to the reported method [33]

	The proposed method	Reported method [33]
NEMI		
Complex GAPI tool		
AGREE tool		
Carbon footprint (kg CO_2_ eq /sample)		
BAGI tool		
RGB12		
NQS		

### Greenness assessment using complex GAPI

A more recent and widely accepted semiquantitative tool. This upgraded version adds the CHEM21 parameters, which account for every stage and process performed prior to the final analytical procedure (Fig S2). This indicates that Complex GAPI assesses all stages of an analytical process, from gathering samples, transportation, and storage to the preparation of samples and early operations before the initiation of the actual analysis. One of the main benefits of Complex GAPI is the usage of open-source software for creating pictograms, rendering it highly accessible. The resulting pictogram switches color from green to yellow to red, offering a clear, measurable evaluation of every step in the analytical procedure, concluding with the final analysis [44, 45]. The low E factor and green pictograms indicate that the suggested method is thought to be environmentally safe. The ecological advantages of these techniques are highlighted by their lower E factor of 1, which denoted less waste production, reduced environmental impact, and increased sustainability conversely to the reported method [33] as outlined in (Table 4).

### Greenness assessment using AGREE

AGREE is one of the best and most popular tools for evaluating the environmental friendliness of analytical methods. By combining all 12 GAC fundamentals, it offers a comprehensive evaluation. The results are displayed in an easy-to-understand, color-coded pictogram, and this tool is flexible enough to apply varying weightings to the different principles. AGREE allows for adjustable scoring by modifying the weights of the 12 GAC principles, which form the basis of its input parameters. Following weight application, the tool produces a final score ranging from 0 to 1. Dark green denotes a score of 1 (great eco-friendliness), while dark red denotes a score of 0 (poor eco-friendliness). The outcome is shown graphically with a score and color in the middle of a clock-like design. [46, 47]. The results, displayed in the graph, demonstrated the proposed method's exceptional environmental adherence. It achieved a high score of 0.75, demonstrating its overall superior ecological aspects conversely to the reported method[33] which scored 0.53, as outlined in (Table 4).

### Analysis of carbon footprint

In green chemistry, measuring the carbon footprint is a common way to evaluate how analytical methods affect the environment, commonly expressed in kilograms of carbon dioxide (CO_2_), this metric quantifies the greenhouse emissions of gases associated with the methods.

[48], unlike to other greenness assessment methodologies, carbon footprint measurements offer a quantitative scale for comparing the environmental effects of different procedures over all stages. This covers things like the use of electricity, the transport of chemicals, and waste generation. As a result, by addressing extra environmental factors that current approaches miss, the carbon footprint analysis improves conventional greenness evaluation tools like NEMI, Complex GAPI, and AGREE. The following typical equation was used to calculate the carbon footprint. Carbon Footprint (kg CO_2_ equivalent) = ∑ Instrument Power (kW) × analysis time (in hours) × Electricity's carbon dioxide emission factor (kg CO_2_/kWh). The study's methodology revealed a carbon footprint of 0.00_2_ kg CO_2_ equivalent per examined sample, conversely to the reported method[33] revealed 0.076 kg CO_2_ per sample as outlined in (Table 4). These methods achieved a lower carbon footprint by reducing electricity consumption—thanks to shorter analysis durations, energy-efficient instrumentation and the avoid of derivatization steps. Additionally, using more environmentally friendly ethanol instead of chlorinated solvents led to a significant reduction in emissions associated with chemical transport, further enhancing the method's environmental sustainability.

### BAGI criteria

Unlike tools that are solely concerned with evaluating greenness, the recently created BAGI metric offers a numerical evaluation of the "blueness" of an analytical procedure. This word describes how well the method works in the actual world, as determined by important practical considerations that affect its efficacy and applicability [49]. By analyzing ten important parameters, the BAGI metric provides a thorough evaluation of the practical applicability or "blueness" of an analytical procedure.These include the analysis type carried out, the number of analytes detected simultaneously, the instrumentation required, Simultaneous sample preparation, the necessities for sample preparation, Samples per h, the reagents and materials needed, the pre-concentration necessity, the level of automation, and sample volume needed. These standards are assessed using a scale of 1 (minimum) to 10 (maximum). Calculating the meaning of the individual scores yields the final BAGI score. Analysis methods with higher BAGI scores are considered more relevant, efficient, and in line with their intended goals. Our method produced an outstanding BAGI score of 90, conversely to the reported method [33] scored 80, demonstrating superior applicability as shown in (Table 4).

### RGB 12

In June 2021, Pawe-Nowak and colleagues developed the RGB 12 tool, a flexible quantitative approach designed to quantify the whiteness of analytical processes by evaluating methods based on the 12 WAC impacts. The RGB 12 algorithm is made up of 12 distinct criteria that are divided into three color groups: red, blue, and green. The green category (G1–G4) focuses on toxicity, reagent and waste production, energy consumption, and the effects on genetic material, animals, and the health of human. The validation requirements, which include aspects such scope of application, accuracy, precision, LOD, and LOQ, are covered by the red category (R1–R4). The blue category (B1–B4) considers practical and economic aspects, involving Cost-effectiveness, time effectiveness, and viability. The three categories' scores—green, red, and blue—are averaged to determine the final "whiteness" score, which indicates how well an approach adheres to WAC standards. [50]. The proposed methods offered an excellent whiteness score of 89.8 conversely to the reported method [33]scored 78.8, as shown in (Table 4), highlighting their significant benefits in terms of environmental sustainability, analytical performance, and the settlement of practical and financial problems.

### The NQS index

The NQS Index is employed in this study as a thorough evaluation tool to gauge how well the methodology complies with sustainable development principles [51]. A comprehensive evaluation of a method's total effectiveness is provided by the integration of three crucial features into one innovative metric: social relevance, analytical performance, and environmental sustainability. The NQS Index (Fig S3) is represented as a triangular pyramid, explaining the balance between need, quality, and sustainability required for reaching top in sustainable analytical chemistry. The method's positioning close to the top of the pyramid suggests that it performs well in all three areas, making it a better choice for sustainable analysis. More information about how the NQS Index is calculated may be found in (Table S5), This outlines the distinct roles played by each dimension and illustrates how these elements work together to form an all-encompassing assessment framework. Our spectrophotometric methods achieved an exceptional 83% NQS Index score, conversely to the reported method [33] scored 41%, as elaborated in (Table 4). This significant difference highlights how well our approach adheres to the United Nations Sustainable Development Goals (UN-SDGs), especially those who work on health (SDG 3), high quality education (SDG 4), cheap and clean energy (SDG 7), and climate action (SDG 13), as outlined in (Table S6).

Ultimately, integrating the NQS Index alongside evaluations of blueness, greenness, and whiteness delivers a holistic perspective on an analytical method’s sustainability impact. The method showcases outstanding performance in environmental impact, practical applicability, and analytical performance, while setting a high bar for advancements in pharmaceutical analysis. The innovative utilization of the NQS Index highlights the method's role in promoting greener scientific practices across the community and aligning innovation with global sustainability goal.

## Conclusion

Three advanced and eco-friendly spectrophotometric chemometric models involving CLS, PLS, and MCR-ALS, were created and validated in this study for the simultaneous quantification of CIN, DOM, and BNZ. A central aspect of this study was the implementation of D-optimal design, an advanced statistical method employed to design an optimal validation dataset. This approach ensures a comprehensive and unbiased assessment of the model’s performance across the entire concentration range, effectively overcoming a usual issue in chemometrics where arbitrary data splitting can result in biased outcomes. The comprehensive evaluations proved the sensitivity, accuracy, and precision of these models, with MCR-ALS serving as the best performer due to its ability to both quantify and qualitatively resolve pure component spectra and demonstrate the smallest and most consistent RMSEP. The study also utilized nine different judgement tools, involving AGREE, Complex GAPI, BAGI, NEMI, RGB12, Spider chart, CF, GSST, and NQS, to determine the method’s greenness, sustainability, whiteness, and analytical efficiency. The outcomes demonstrate that the proposed methods perform exceptionally well regarding environmental sustainability, analytical efficiency, and practicality.

## Supplementary Information

Additional file1 (DOCX 680 KB)

## Data Availability

Datasets generated or analyzed during this study are available from the corresponding author upon reasonable request.
