# Supplementary material for: MATLAB-candexch algorithm-enhanced UV spectrophotometric-chemometric models for green, blue, and white determination of cinnarizine, domperidone, and carcinogenic impurity in pharmaceuticals: NQS assessment and UN-SDGs integration
Source: BMC Chem. 2026 Apr 12;20(1):86. doi: 10.1186/s13065-026-01779-0 (PMC13085644; doi:10.1186/s13065-026-01779-0)
Supplement: Supplementary file 1 — Additional file1 (DOCX 680 KB) [file 13065_2026_1779_MOESM1_ESM.docx]

**Supplementary Figures**


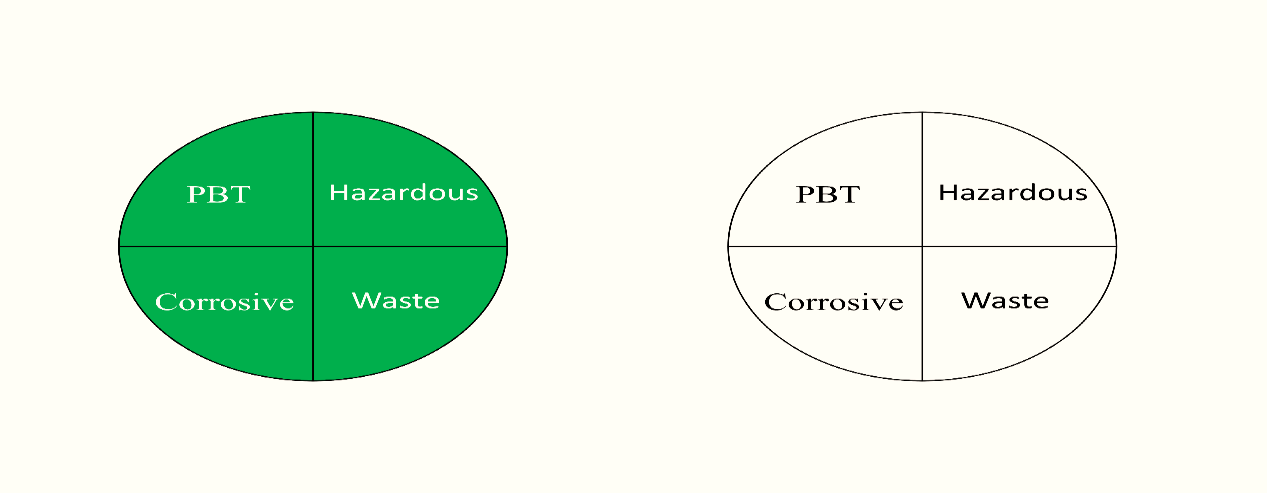


**Fig. S1.** Typical NEMI pictograms


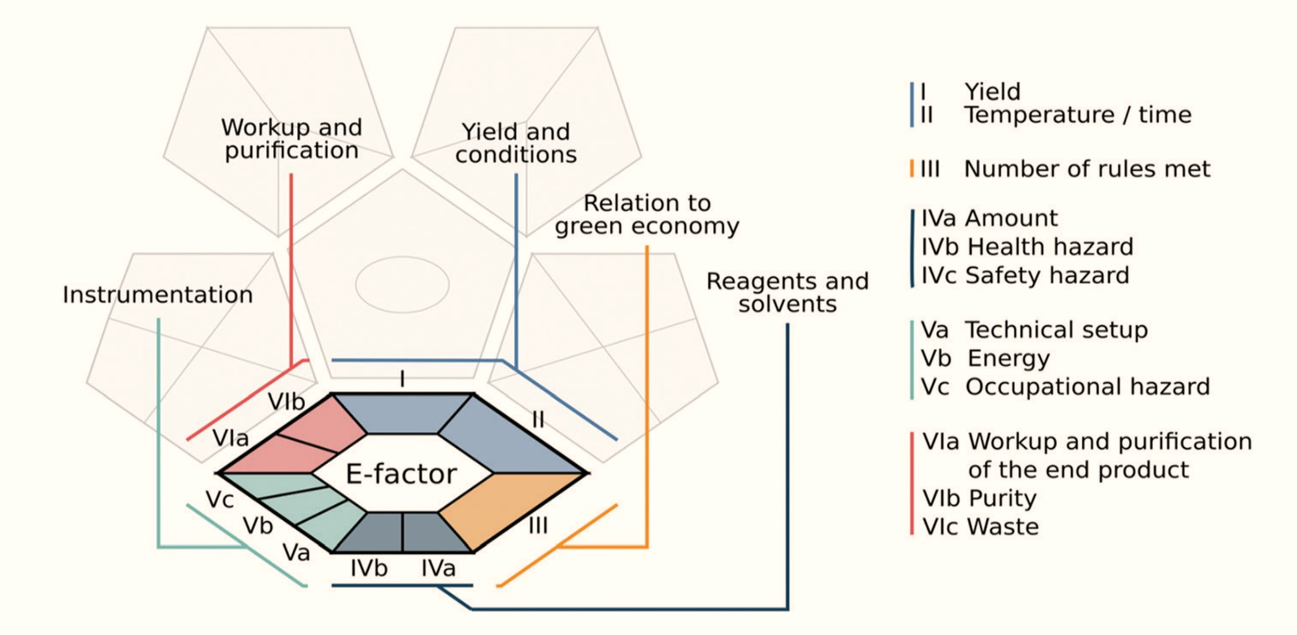


**Fig. S2.** The ComplexGAPI pictogram, with the original GAPI pictogram greyed out in the background, and particular fields of the added hexagonal glyph grouped and colour-coded for clarity.


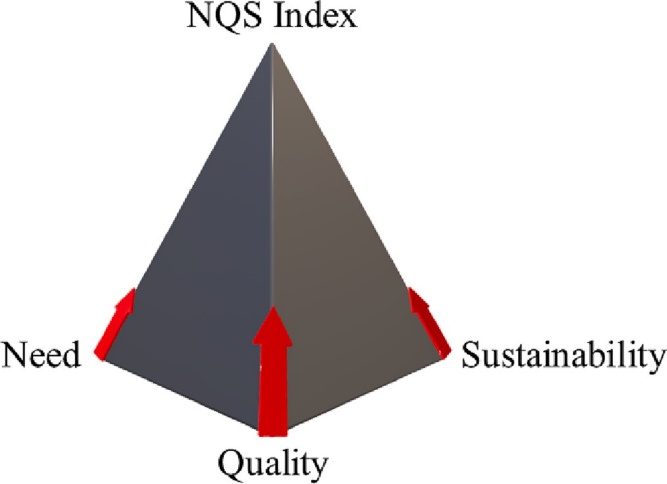


**Fig. S3.** Triangular pyramid representing analytical chemistry for sustainability concept with the Need, Quality, and Sustainability (NQS) index.

**Supplementary Tables**

**Table S1:** Greenness Index values for solvents of proposed and reported methods attained by spider diagram for assessment of the greenness index

| **Evaluation parameters** | **Health Impact** | **General Properties** | **Fire Safety** | **Odor** | **Stability** | **Score average** |
| --- | --- | --- | --- | --- | --- | --- |
| **Ethanol score** | 2.63 | 0.69 | 0.61 | 0 | 2.71 | 1.33 |
| Available information% | 100 | 87.50 | 100 | 100 | 85.71 | 97.50 |
| **Ethyl acetate score** | 2.13 | 1.78 | 0.17 | 0 | 3.14 | 1.44 |
| Available information% | 100 | 87.50 | 66.67 | 100 | 85.71 | 87.98 |
| **Hexane score** | -0.06 | -0.88 | -1.17 | -3.00 | 0.57 | -0.91 |
| Available information% | 75 | 68.75 | 100 | 100 | 71.43 | 83.04 |
| **Chloroform score** | -1.25 | -0.26 | 2.00 | -3.00 | -0.14 | -0.06 |
| Available information% | 81.25 | 87.50 | 100 | 100 | 57.14 | 85.18 |
| **Methanol score** | -0.75 | -1.06 | 4.33 | -3.00 | -0.14 | -0.12 |
| Available information% | 68.75 | 75 | 100 | 100 | 100 | 88.75 |
| **Acetonitrile score** | 0.50 | 0.00 | 0.17 | -1.00 | -1.14 | -0.30 |
| Available information% | 62.50 | 87.50 | 100 | 100 | 71.43 | 84.29 |

**Table S2:** Cosine similarity analysis of MCR-ALS recovered profiles vs standard spectra.

| **Component** | **Cosine Similarity** | **Peak Alignment** | **Intensity Ratio*** | **Key Observations** |
| --- | --- | --- | --- | --- |
| DOM | 0.992 | Excellent | 0.940 | Minor intensity difference at 285 nm peak |
| CIN | 0.988 | Good | 0.910 | Noticeable deviation in peak shape at 240-260 nm region |
| BNZ | 0.994 | Excellent | 0.970 | Slight difference in main peak intensity at 250 nm |

***Intensity Ratio = Peak intensity of recovered spectrum / Peak intensity of standard spectrum**

**Table S3:** Comprehensive Rotational Ambiguity Assessment

| **Component** | **AFS (%)** | **Avg RFS (AU)** | **Max RFS (AU)** | **Critical λ (nm)** | **Impact on Quantitation*** |
| --- | --- | --- | --- | --- | --- |
| DOM | 5.8 | 0.009 | 0.021 | 285 ± 2 | Minimal (< 1.7%) |
| CIN | 8.2 | 0.014 | 0.027 | 253 ± 2 | Moderate (< 2.9%) |
| BNZ | 6.5 | 0.011 | 0.023 | 252 ± 2 | Moderate ( < 2.1 %) |

***** **Impact on quantitation refers to the potential relative error in concentration estimates due to rotational ambiguity.**

**Table S4:** One-way ANOVA statistical analysis of the results obtained by applying the proposed chemometric methods for determining DOM and CIN in the dosage form by the proposed methods and the reported method [28] within a 95% confidence limit.

| **Component** |  | **Sum of squares** | **df** | **Mean square** | **F (3.2388)^a^** | **P value** |
| --- | --- | --- | --- | --- | --- | --- |
|  | | | | | | |
| **DOM** | **Between Groups** | 13.844 | 3 | 4.614 | 2.730 | 0.999 |
|  | **Within Groups** | 27.041 | 16 | 1.690 | -------- | -------- |
|  | **Total** | 40.885 | 19 | -------- | -------- | -------- |
|  | | | | | | |
| **CIN** | **Between Groups** | 4.233 | 3 | 1.411 | 1.335 | 0.999 |
|  | **Within Groups** | 16.911 | 16 | 1.057 | -------- | -------- |
|  | **Total** | 21.145 | 19 | -------- | -------- | -------- |

^a^ Figures in parentheses represent the corresponding critical value of F at P < 0.05.

**Table S5:** Calculations of the NQS Index.

|  | **The proposed method** | **The reported method [33]** |
| --- | --- | --- |
| **Need** | **100** | **25** |
| **Quality** | **90** | **78.8** |
| **Sustanability** | **59** | **18** |
| **NQS Index** | **83** | **41** |

**Table S6:** Alignment of eco-friendly chemometric spectrophotometric technique with UN-SDGs 3, 4, 5, 7, 11, 12,13,14, 15, and 17

| **SDG** | **Goal** | **Application(s)** |
| --- | --- | --- |
| 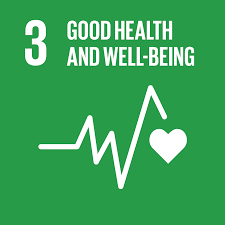 | **Good health and well-being** | - Enhances laboratory quality control for improved drug analysis. - Reduces errors in pharmaceutical quantification - Expands access to high-quality analysis in resource-limited settings |
| 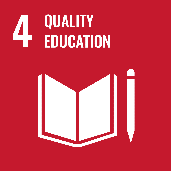 | **Quality Education** | - Advances analytical chemistry education through sustainable spectrophotometric techniques - Promotes interdisciplinary learning in chemistry and sustainability |
| 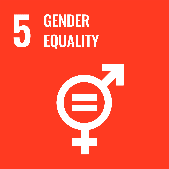 | **Gender Equality** | - Fosters inclusive research environments, promoting gender diversity in analytical chemistry - Supports equitable participation in research teams |
| 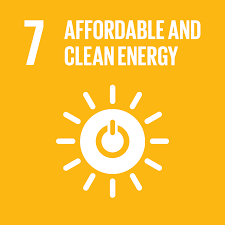 | **Affordable and clean energy** | - Utilizes energy-efficient spectrophotometric methods - Integrates sustainable energy solutions in analytical processes |
| 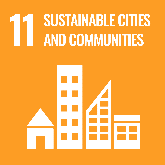 | **Sustainable Cities and Communities** | - Implements eco-friendly analytical techniques for urban environmental monitoring - Contributes to resource-efficient community laboratories |
| 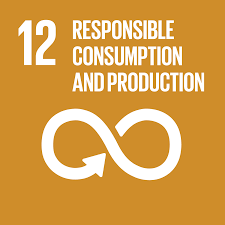 | **Responsible consumption and production** | - Employs green solvents and minimizes hazardous waste in analytical procedures - Optimizes resource use in chemical analysis |
| 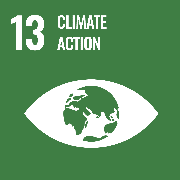 | **Climate Action** | - Reduces carbon footprint of analytical processes - Minimizes energy consumption and harmful emissions in laboratory operations |
|  | **Life below water** | - Supports aquatic ecosystem analysis with minimal environmental impact - Enhances water quality monitoring using sustainable methods |
|  | **Life on land** | - Facilitates eco-friendly terrestrial ecosystem analysis - Minimizes environmental impact in soil and plant tissue studies |
|  | **Partnerships for the goals** | - Promotes international collaboration in sustainable analytical chemistry - Facilitates knowledge exchange between researchers globally |
